# Assessing Second‐Line Treatment Strategies and Outcomes in Epidermal Growth Factor Receptor (EGFR) Oncogene‐Driven Stage IV Non‐Small Cell Lung Cancer, Following a First‐Line EGFR‐TKI Therapy

**DOI:** 10.1002/cnr2.70428

**Published:** 2025-12-17

**Authors:** Meghana Maddula, Annelise Decaria, Bea Brown, John Simes, Michael Boyer, Venessa Chin

**Affiliations:** ^1^ University of New South Wales Sydney Australia; ^2^ The Kinghorn Cancer Centre Darlinghurst Australia; ^3^ St Vincent's Hospital Sydney Australia; ^4^ NHMRC Clinical Trials Centre The University of Sydney Sydney New South Wales Australia; ^5^ The University of Sydney Sydney New South Wales Australia; ^6^ Chris O'Brien Lifehouse Sydney New South Wales Australia; ^7^ The Garvan Institute of Medical Research Sydney Australia; ^8^ The Kinghorn Cancer Centre St Vincent's Hospital Sydney Australia

**Keywords:** EGFR, NSCLC, real‐world data, TKI

## Abstract

**Background:**

Oncogene‐driven metastatic non‐small cell lung cancer (mNSCLC) is associated with poor survival outcomes, despite advancements in first‐line tyrosine kinase inhibitor (TKI) therapies.

**Aims:**

This study aimed to characterise second‐line treatment strategies for epidermal growth factor receptor (EGFR)‐driven mNSCLC, and to evaluate associated clinical outcomes using real‐world data.

**Materials:**

The EnRICH study prospectively collected clinical data on patients diagnosed with lung cancer from NSW, Australia. Patients with EGFR‐driven mNSCLC who received second‐line therapy following progression on first‐line EGFR‐TKI were included in this analysis. Outcomes assessed included progression‐free survival (PFS2) and overall survival (OS), measured from second‐line treatment.

**Results:**

Of 2000 patients with newly diagnosed lung cancer, between 2016 and 2021, 1720 had NSCLC and 679 had metastatic disease. Among these, 647 underwent molecular testing, with 133 harboring an EGFR mutation. Of these 133, 80 patients received first‐line TKI therapy. From this group, 68 who subsequently received second‐line treatment following disease progression on first‐line TKI were included in this analysis. Of these 68 patients, 77% (*n* = 52/68) were treated with a first‐ or second‐generation TKI (Erlotinib: *n* = 39, Gefitinib: *n* = 7, Afatinib: *n* = 6) in the first‐line setting. The remaining 16 patients received the third‐generation TKI Osimertinib. Of the total 68 patients in this cohort, in the second‐line setting, 45 patients underwent a change in their systemic therapy, and the remaining 23 patients received radiotherapy either with cessation of first‐line TKI (*n* = 11) or in addition to continuation of first‐line TKI (*n* = 12). Of the 45 patients who underwent a change in their systemic therapy, 32 received systemic therapy alone in the second‐line setting and 13 received radiotherapy in addition to a change in systemic therapy. Of these 45 patients, 33 received a TKI, with the majority receiving Osimertinib (*n* = 28). Median PFS2 was 3.0 months (95% CI: 1.32–5.49), with no significant difference between patients treated with second‐line TKI and those receiving other therapies (*p* = 0.24). Median OS was 15.0 months (95% CI: 13.24–23.66) and similarly did not differ significantly between second‐line TKI and alternative treatment strategies (*p* = 0.17).

**Conclusions:**

Using real‐world data from EnRICH, this represents the largest retrospective analysis of this cohort in Australia and largely predates first‐line Osimertinib use. Findings reveal a trend toward second‐line Osimertinib use, even after first‐line TKI, without significant differences in survival. There, however, remains significant heterogeneity in approaches. The lack of robust second‐line data, however, highlights the need for investigating novel therapeutic approaches.

## 1 Introduction

Non‐small cell lung cancer (NSCLC) accounts for up to 85% of all lung cancer diagnoses and remains a leading cause of cancer‐related mortality [1, 2, 3, 4]. The majority of patients present with advanced disease and experience poor outcomes, with 5‐year overall survival (OS) rates as low as 5% [1, 2, 3, 4]. Molecular testing and next generation sequencing (NGS) for oncogene‐driven mutations, including epidermal growth factor receptor (EGFR) gene mutations, are now the standard of care for the diagnosis and management of metastatic NSCLC [5, 6].

Between 10% and 30% of NSCLCs harbor EGFR mutations [5, 6, 7], occurring more frequently in female, nonsmoking individuals of Asian descent [8, 9]. This genetic aberration provides a molecular target for therapeutic intervention, and the role of oral targeted therapy with EGFR tyrosine kinase inhibitors (TKIs) in previously untreated EGFR oncogene‐driven metastatic NSCLC is now well established [10, 11, 12, 13, 14, 15, 16, 17]. First‐generation EGFR‐TKIs such as Erlotinib and Gefitinib have been available through government‐funded access in Australia since 2004 and 2008, respectively, with the second‐generation EGFR‐TKI Afatinib becoming available in 2018 [18, 19] (Table S1). Osimertinib, a third‐generation EGFR‐TKI, was subsequently approved for second‐line use in 2018 and first‐line use in 2021 in Australia [18, 19], further shaping prescribing practices and improving survival outcomes [17, 18, 19] (Table S1).

Intrinsic or acquired resistance and disease progression are inevitable, subsequently impacting patient outcomes and prognosis, and the optimal approach in the second‐line setting remains unclear. In cases of isolated progression, local therapies such as surgery or radiotherapy can be considered [20, 21, 22, 23]. If systemic therapy is warranted, there is significant heterogeneity in approaches, ranging from subsequent EGFR‐TKI therapy, various chemotherapy agents, immunotherapy, emerging bi‐specific agents, and pursuit of clinical trials [24, 25, 26, 27, 28, 29, 30, 31, 32]. Resistance to EGFR‐TKIs, particularly with early‐generation EGFR‐TKIs and in patients with T790M mutations, is now widely recognized [33, 34], The scarcity of clinical trial data to guide subsequent lines of therapy highlights the importance of investigating real‐world data.

The Embedding Research (and evidence) in Cancer Healthcare (EnRICH) program [35] has prospectively recruited a clinical cohort of newly diagnosed lung cancer patients across metropolitan and regional New South Wales, Australia since 2016. This study leveraged real‐world data from the EnRICH database to investigate real‐world first‐ and second‐line treatment strategies in EGFR oncogene‐driven NSCLC, patterns of disease progression, and compared survival outcomes to guide treatment strategies moving forward.

## 2 Materials and Methods

### 2.1 Study Design and Participants

The EnRICH database prospectively collects demographic, disease, treatment, and outcome data for patients with newly diagnosed lung cancer presenting to nine specialist cancer centers in metropolitan and regional New South Wales, Australia. This observational study included data for a cohort of 2000 patients diagnosed with lung cancer between September 2016 and October 2021; 1720 patients were identified as having NSCLC. Patients with EGFR oncogene‐driven metastatic NSCLC (mNSCLC) who had received EGFR‐TKI therapy in the first‐line setting, and required subsequent therapy in the second‐line setting due to disease progression were identified for analysis (Figure S1). Patients with EGFR‐mutant NSCLC who did not receive a first‐line EGFR‐TKI were not included in this analysis, as the study was designed to focus on outcomes in those treated with first‐line EGFR‐TKI. Data cutoff was March 31, 2024.

In this study, line of treatment in this study referred solely to the sequence in which therapies were delivered, and not to the drug class, modality, or generation of treatment. First‐line therapy was restricted to EGFR‐TKI, as this was an explicit inclusion criterion for cohort selection. No patients in this cohort received chemotherapy, immunotherapy, or combined‐modality treatment in the first‐line setting. Second‐line therapy was defined as the subsequent treatment commenced after documented progression on first‐line therapy, regardless of whether this consisted of a different EGFR‐TKI, chemotherapy, immunotherapy, or combined‐modality treatment. For the purposes of this study, “second‐line systemic therapy” was defined as the initiation of a new systemic agent following documented disease progression on first‐line EGFR‐TKI therapy. This included substitution of the first‐line EGFR‐TKI with another EGFR‐TKI, addition of a new EGFR‐TKI to ongoing treatment, or initiation of alternative non‐TKI systemic agent, either alone or in addition to radiotherapy. Patients who continued their first‐line EGFR‐TKI without any change in systemic agent, were not classified as having received “second‐line systemic therapy.”

Patient characteristics including demographics (age and sex), medical history (smoking status), and Eastern Cooperative Oncology Group (ECOG) performance status (PS) were examined. Data was also extracted on disease characteristics (histopathology, oncogene‐driven mutations, and sites of metastases), treatment characteristics (line of treatment, type, duration, and reason for cessation), and patterns of response (best response, progression, and management). Subgroups were evaluated based on treatment type.

This study was approved as a sub‐study (protocol number X16‐447, HREC/16/RPAH/634, 2019/ETH06379), as part of the EnRICH Research Program.

### 2.2 End Points

Efficacy endpoints included progression‐free survival (PFS) and OS. PFS1 was assessed from the date of diagnosis, and PFS2 was assessed from the date of second‐line treatment commencement to the date of disease progression as determined by individual investigator assessment, or censoring. OS was evaluated from the commencement of second‐line treatment to the date of last follow‐up or death (obtained from medical records and monthly linkage with death notifications recorded in the NSW registry of births, deaths, and marriages). Patients who were lost to follow‐up had their data censored at the time of the last follow‐up visit or the date of dataset closure. PFS2 and OS were evaluated in the overall cohort and also stratified by treatment type. Management of oligometastatic disease was also examined, with oligometastatic disease and oligoprogression defined as progression in fewer than three metastatic sites across one or two organ systems as defined by Hellman and Weichselbaum and the ESTRO–ASTRO consensus guidelines [36, 37, 38]. Patients not meeting these criteria were classified as having systemic disease progression.

### 2.3 Statistical Analysis

Descriptive analysis of baseline characteristics and treatment strategies was conducted. Subgroup analysis was conducted based on first‐ and second‐line treatment type. Differences across subgroups were assessed using the Wilcoxon rank‐sum test or Chi‐square test for continuous variables and Fisher's exact test for categorical variables.

PFS and OS curves were generated using the Kaplan–Meier method. Differences between groups were assessed using the log‐rank test, with a two‐sided *p*‐value < 0.05 considered statistically significant. The *p*‐values displayed on Kaplan–Meier curves were derived from the log‐rank test. Cox proportional hazards models were applied to estimate hazard ratios (HRs) with 95% confidence intervals, and the associated *p*‐values reported correspond to the HRs. Exploratory Cox proportional hazards models were performed to assess associations between age, ECOG performance status, and survival outcomes.

All statistical analyses were performed using R version 4.4.1.

## 3 Results

### 3.1 Patient and Disease Characteristics

Of the 2000 patients with newly diagnosed lung cancer enrolled in the EnRICH program between September 2016 and October 2021, 1720 had NSCLC, and 679 had metastatic disease. Of the 679 patients with mNSCLC, 647 underwent molecular testing, and 133 were identified to have an EGFR oncogene‐driven mutation. Data were not available on the subtype of EGFR mutation or T790M mutation status for review. Among the 133 patients with EGFR oncogene‐driven mutations, 80 patients within this group received first‐line EGFR‐TKI therapy. All of these patients received EGFR‐TKI as monotherapy. Of these 80 patients, 68 experienced disease progression and subsequently received second‐line treatment, comprising systemic therapy, radiotherapy, or combined‐modality therapy, and were therefore included in this analysis. A schematic of the patient inclusion process is provided in Figure S1. These 68 patients formed the study cohort and served as the denominator for primary analyses of first‐line and second‐line treatment strategies. Detailed breakdowns of treatment approaches (e.g., use of radiotherapy, systemic therapy type) are provided in the corresponding results sections and figures. Data regarding other patients within the EnRICH program, including those who did not progress to second‐line therapy, were not included in this analysis.

The median follow‐up period was 12 months.

The median age at diagnosis was 63 years old (range 57–73), 63.2% (*n* = 43/68) were female and 94.1% (*n* = 64/68) had an ECOG performance status of 0 or 1 (Table 1). The most common country of birth was Asia (51.5%, *n* = 35/68). The most common histopathology was adenocarcinoma (95.6%, *n* = 65/68).

**TABLE 1:** Patient and disease characteristics.

Patient and disease characteristics (*n* = 68)	
Median age	63 (57–73)
Sex	
Female	43 (63.2%)
Male	25 (36.8%)
Country of birth	
Asia	35 (51.5%)
Australia	19 (27.9%)
Europe	13 (19.1%)
Americas	1 (1.5%)
ECOG^a^ performance status	
0	36 (52.9%)
1	28 (41.1%)
2	1 (1.5%)
3	3 (4.4%)
Smoking status	
Current	2 (2.9%)
Ex‐smoker (≥ 5 pack‐year history)	22 (32.4%)
Never	37 (54.4%)
Passive‐smoker (nonsmoker lived in house with smoker ≥ 5 years)	7 (10.3%)
Histopathology	
Adenocarcinoma	65 (95.6%)
Other	2 (2.9%)
Unknown	1 (1.5%)
PD‐L1 score^b^	
100%	0 (0)
≥ 50%	11 (16.2%)
1%–49%	31 (45.6%)
< 1%	12 (17.7%)
Unknown	14 (20.6%)

^a^ Eastern Cooperative Oncology Group.

^b^ Programmed death‐ligand 1.

### 3.2 First‐Line Treatment Strategies

Of the 68 patients analyzed in this study, all received first‐line EGFR‐TKI therapy. All treatments in the first‐line setting consisted of EGFR‐TKI therapy alone, with no patients receiving concurrent radiotherapy or other local therapy. Among the total cohort, 46 patients (67.7%, *n* = 46/68) were treated with first‐generation EGFR‐TKIs (Erlotinib: *n* = 39, Gefitinib: *n* = 7), 6 patients (8.8%, *n* = 6/68) received the second‐generation EGFR‐TKI Afatinib, and 16 patients (23.5%, *n* = 16/68) received the third‐generation TKI Osimertinib (Table 2). The distribution of first‐line treatment by EGFR‐TKI generation is additionally shown in Figure S2.

**TABLE 2:** Treatment strategies.

First‐line treatment (*n* = 68)	
First‐generation EGFR‐TKI	
Erlotinib	39/68 (57.4%)
Gefitinib	7/68 (10.3%)
Second‐generation EGFR‐TKI	
Afatinib	6/68 (8.8%)
Third‐generation EGFR‐TKI	
Osimertinib	16/68 (23.5%)

Second‐line treatment (*n* = 68)	
Management strategy	
Systemic therapy alone	32/68 (47.1%)
Radiotherapy + change in second‐line systemic therapy	13/68 (19.1%)
Radiotherapy + cessation of first‐line EGFR‐TKI (aka radiotherapy alone)	11/68 (16.2%)
Radiotherapy + continuation of first‐line EGFR‐TKI	12/68 (17.6%)
Radiotherapy sites (*n* = 36)	
Brain	11/36 (30.6%)
Lung	5/36 (13.9%)
Other sites	20/36 (55.6%)

### 3.3 Patterns of Progression and Survival Outcomes Following First‐Line EGFR‐TKI

**FIGURE 1:**
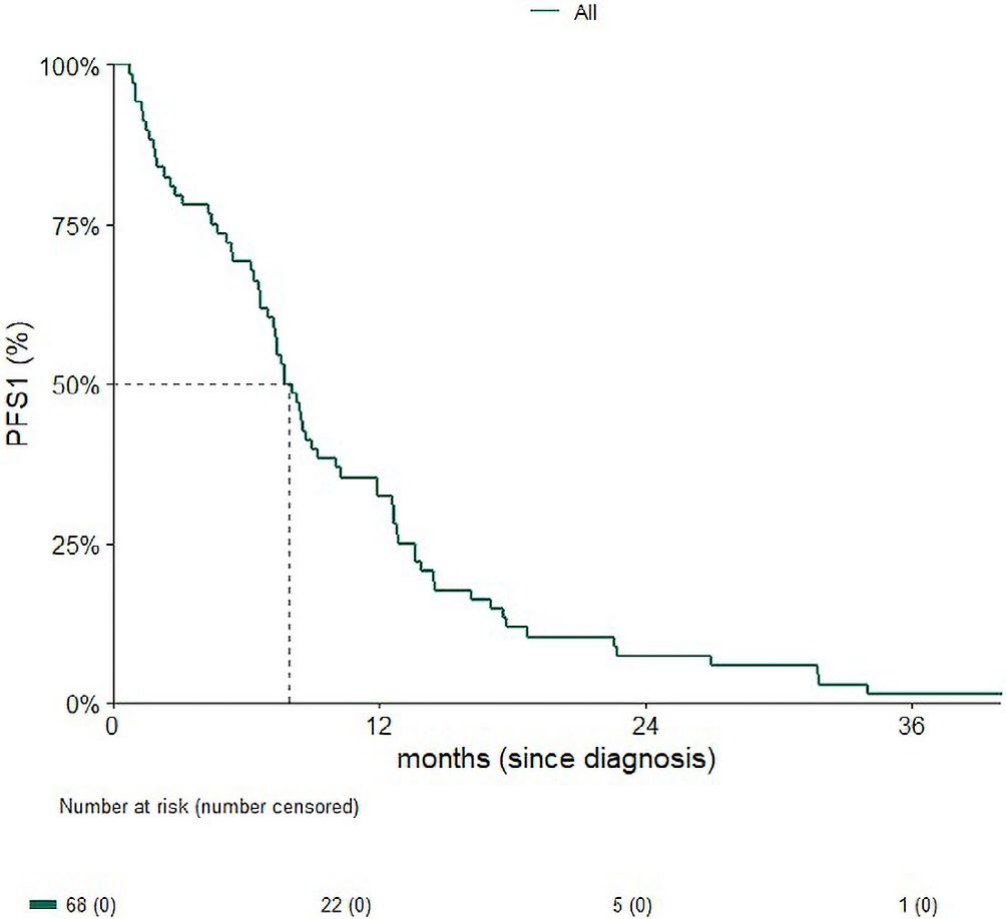
Progression‐free survival 1.

With a median follow‐up of 12 months, the median PFS1 for the overall cohort was 7.93 months [95% CI: 7.0–10.23] (Figure 1). In an exploratory analysis, age and ECOG performance status were not significantly associated with PFS1. The HR for age was 0.99 [95% CI: 0.96–1.01], and for ECOG 3 compared with ECOG 0, the HR was 1.78 [95% CI: 0.53–59.4].

In this cohort, 50% (*n* = 34/68) developed oligoprogression on first‐line treatment. Among these, 50% (*n* = 17/34) had progression confined to the lung. Other sites of oligoprogression included brain (26.5%, *n* = 9/34), bone (20.6%, *n* = 7/34), and adrenal gland (2.9%, *n* = 1/34).

Of the remaining 50% (*n* = 34/68) patients with systemic disease progression, 67.7% (*n* = 23/34) experienced progressive disease within the lung. Other sites of progression included bone (41.2%, *n* = 14/34), brain (29.4%, *n* = 10/34), liver (11.8%, *n* = 4/34), adrenal gland (2.9%, *n* = 1/34), and other sites of disease (50%, *n* = 17/34).

### 3.4 Second‐Line Treatment Strategies

All 68 patients included in the study received both first‐line EGFR‐TKI and subsequent second‐line treatment following disease progression. The median time from disease progression to commencement of second‐line treatment was 37 days (range: 0–558 days).

Of these 68 patients, 45 (66.2%) underwent a change in systemic therapy in the second‐line setting, encompassing both those who received systemic therapy alone (*n* = 32) and those treated with radiotherapy to the site of metastases in addition to systemic therapy (*n* = 13). The remaining 23 patients received radiotherapy either with cessation of first‐line EGFR‐TKI (*n* = 11) or in addition to continuation of first‐line EGFR‐TKI (*n* = 12).

Among the 32 patients (47.1%, *n* = 32/68) who underwent a change in systemic therapy alone in the second‐line setting, the majority were given an alternative EGFR‐TKI (*n* = 23); 17 treated with Osimertinib, 2 with Erlotinib, 2 patients who were initially treated with Gefitinib changed EGFR‐TKI due to toxicity (Erlotinib: *n* = 1, Osimertinib: *n* = 1), and 2 patients cycled between Gefitinib and Osimertinib as part of the Oscillate clinical trial [39]. The remaining 9 patients received chemotherapy‐based regimens (Figure S3).

Radiotherapy was administered in the remaining 36 patients (52.9%, *n* = 36/68) in the cohort. Of these, 11 (30.6%) received radiotherapy alone with cessation of first‐line EGFR‐TKI,12 (33.3%) received radiotherapy with continuation of first‐line EGFR‐TKI, and 13 (36.1%) received radiotherapy in conjunction with a change in systemic therapy (Figure S4). Among the 13 patients, 10 changed treatment to Osimertinib and 3 received chemotherapy‐based regimens (Table 2; Figures S3 and S4).

Therefore, in total, 33 (48.5%, *n* = 33/68) patients underwent a change in systemic therapy in the second‐line setting to receive an alternative EGFR‐TKI, either as systemic therapy alone (*n* = 23/68) or in addition to receiving radiotherapy (*n* = 10/68). Pertinently, although T790M mutation status was not available for review in this study, it should be noted that in Australia access to Osimertinib in the second‐line setting requires the presence of a T790M mutation [18, 19] (Table 2; Figure S3).

Detailed information on the clinical decision‐making processes involved in management at progression was not captured by this dataset and therefore not available for analysis. The observed variation in the application of radiotherapy, change in EGFR‐TKI, or continuation of EGFR‐TKI likely reflects the inherent heterogeneity of real‐world practice rather than adherence to predefined treatment guidelines.

#### 3.4.1 Radiotherapy Sites

Of the 68 patients included in this study, 36 (52.9%) received radiotherapy to sites of progressive disease, including the brain (30.6%, *n* = 11), lung (13.9%, *n* = 5), and other metastatic sites (55.6%, *n* = 20) (Table 2). Radiotherapy strategies are also visually represented in Figure S4.

#### 3.4.2 Management of Oligoprogression

Of the 34 patients with oligoprogressive disease, 19 underwent a change in systemic therapy alone, and 15 were treated with radiotherapy in the second‐line setting. Of these 15 patients, 7 continued their first‐line EGFR‐TKI, 3 changed to Osimertinib, 1 changed to a chemotherapy‐based regimen, and 4 received no additional systemic therapy.

### 3.5 Survival Outcomes Following Second‐Line Treatment

#### 3.5.1 PFS

**FIGURE 2:**
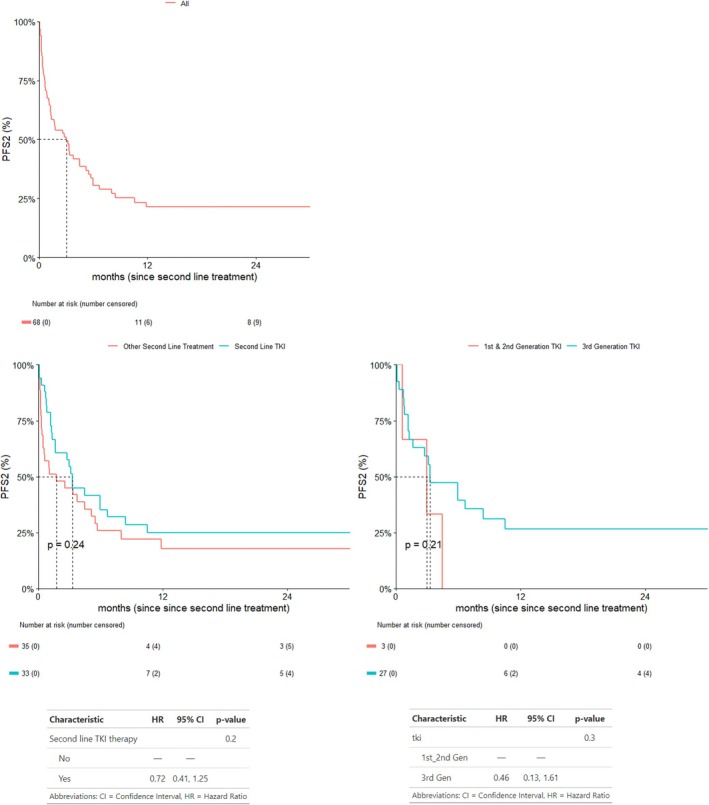
Progression‐free survival 2.

With a median follow‐up of 12 months, the median PFS2 was 3.0 months [95% CI: 1.32–5.49] (Figure 2).

PFS2 was longer in patients who received a second‐line EGFR‐TKI, irrespective of whether radiotherapy was also used, when compared with other treatment strategies [3.29 months (95% CI: 1.64–8.42) vs. 1.74 months (95% CI: 0.49–5.49), *p* = 0.24] [HR 0.72; 95% CI: 0.41–1.25; *p* = 0.2] (Figure 2).

PFS2 was also longer in patients treated with a third‐generation EGFR‐TKI in the second‐line setting, when compared with first‐ or second‐generation EGFR‐TKIs [3.32 months (95% CI: 1.68–NA) vs. 2.99 months (95% CI: 0.62–NA), *p* = 0.21] [HR 0.46; 95% CI: 0.13–1.61; *p* = 0.3]; however, this was also not statistically significant. One patient who switched between generations of EGFR‐TKI due to toxicity (Gefitinib to Osimertinib), and 2 patients who alternated between Gefitinib and Osimertinib as part of the Oscillate clinical trial [39] were excluded from this analysis, as these treatment courses did not represent progression‐directed second‐line therapy and could obscure the interpretation of PFS2 by introducing non‐comparable treatment dynamics, resulting in a total of 30 patients included in this analysis.

In an exploratory analysis, age and ECOG performance status were not significantly associated with PFS2. The HR for age was 0.99 [95% CI: 0.96–1.01], and for ECOG 3 compared with ECOG 0, the HR was 1.61 [95% CI: 0.48–5.38].

#### 3.5.2 OS

At the time of data cutoff, 54 (79.4%) patients had died.

**FIGURE 3:**
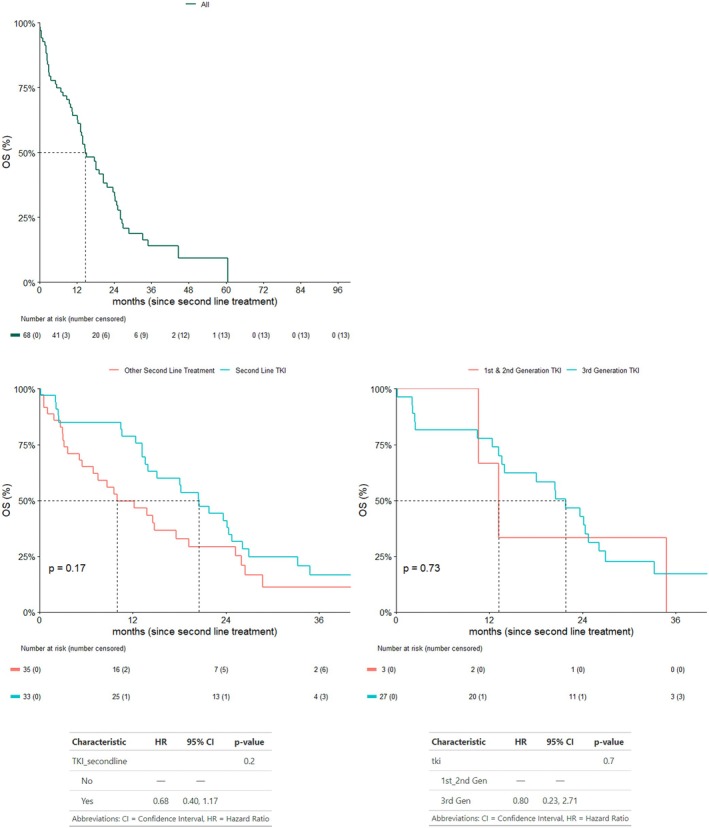
Overall survival 2.

With a median follow‐up of 12 months, the median OS in this cohort was 14.78 months [95% CI: 13.24–23.66] (Figure 3). OS was longer in patients treated with a second‐line EGFR‐TKI, irrespective of whether radiotherapy was also used, when compared with other treatment strategies [20.57 months (95% CI: 14.0–26.12) vs. 9.99 months (95% CI: 6.9–25.26), *p* = 0.17] [HR 0.68; 95% CI: 0.40–1.17; *p* = 0.2], and in patients treated with a third‐generation EGFR‐TKI when compared with earlier‐generation EGFR‐TKIs [21.85 months (95% CI: 14.00–26.94) vs. 13.24 months (95% CI: 10.64–NA), *p* = 0.73] [HR 0.80; 95% CI: 0.23–2.71; *p* = 0.7]; however, these findings were not statistically significant (Figure 3). Applying the exclusion criteria used in the PFS2 analysis, when stratifying OS by generation of EGFR‐TKI, 1 patient who switched between EGFR‐TKI generations due to toxicity (Gefitinib to Osimertinib) and the 2 patients who were enrolled in the Oscillate clinical trial [39], were again excluded, resulting in a total of 30 patients being included in this analysis.

In an exploratory analysis, age and ECOG performance status were not significantly associated with OS. The HR for age was 0.99 [95% CI: 0.97–1.02], and for ECOG 3 compared with ECOG 0, the HR was 2.72 [95% CI: 0.81–9.12].

## 4 Discussion

This study offers real‐world insights into the treatment strategies and outcomes for patients with EGFR oncogene‐driven mNSCLC, reflecting the largest retrospective analysis of this cohort in Australia, facilitated through the EnRICH program [35]. This cohort largely predates the routine use of Osimertinib as a first‐line therapy, providing a unique perspective on treatment patterns. Prior to the availability of Osimertinib, the absence of effective second‐line therapies represented a major clinical challenge. Now that Osimertinib is established in the first‐line setting and is expanding into adjuvant (ADAURA) [40] and stage III (LAURA) [41] settings, progression following its use has reexposed this therapeutic gap and also highlighted the need for effective alternative later‐line therapeutic options.

Our study highlights the significant heterogeneity in second‐line treatment approaches, reflecting the lack of standardized strategies and limited treatment options. Given the timing of our data, most patients in the first‐line setting received first‐ and second‐generation EGFR‐TKIs (Erlotinib, Gefitinib, and Afatinib). The median PFS1 in this cohort was 7.93 months, lower than the reported 10–11 months in pivotal clinical trials [10, 11, 12, 13, 14, 15, 16, 17]. This likely reflects differences in patient characteristics, such as poorer ECOG performance status, as well as additional real‐world factors such as delays in treatment initiation and less intensive monitoring or compliance issues. In the second‐line setting, there was clear preferential use of Osimertinib, with only limited use of chemotherapy/immunotherapy, reflecting evolving prescribing practices in Australia following the second‐line availability of Osimertinib in 2018 [18, 19]. In this retrospective study, management decisions were not protocolized, and the choice between changing to alternative systemic therapy versus continuation of first‐line EGFR‐TKI or incorporation of radiotherapy was determined by treating clinicians in the context of individual disease pattern, patient functional status, and treatment access. This variation further highlights the complexity of real‐world decision‐making, which contrasts with the uniform pathways seen in clinical trials.

Additionally, our study provides insight into current treatment strategies in comparison with available trial data. AURA3 [24] evaluated Osimertinib as a second‐line option for T790M‐positive patients versus chemotherapy, demonstrating a median PFS of 10.1 months and OS of 26.8 months, while IMpower150 [25] assessed carboplatin, paclitaxel, and bevacizumab with and without atezolizumab, reporting a median OS of 27.8 months in atezolizumab‐containing arms (Table S2). In contrast, our real‐world cohort demonstrated inferior outcomes, with a median PFS2 of 3.0 months and OS of 14.78 months in the overall cohort. When stratified by treatment type, median OS was 20.57 months for patients receiving second‐line EGFR‐TKI therapy compared with 9.99 months for those receiving other treatments. These discrepancies likely reflect variation between heterogeneous real‐world and clinical trial populations, alongside additional real‐world factors such as variability in access to therapies, limited Osimertinib use in the second‐line setting, inconsistent treatment adherence, and less‐standardized monitoring and follow‐up practices. Outcomes may also have been influenced by the progression‐enriched nature of our real‐world cohort, in which only patients who had failed first‐line therapy and proceeded to subsequent treatment were included, inherently biasing toward a poorer‐prognosis population. Moreover, as this was a retrospective study, progression was assessed by treating investigators rather than blinded central review, introducing heterogeneity in survival outcome assessment. Finally, it should be acknowledged that this cohort was derived entirely from New South Wales, Australia, where access to therapies was determined by national reimbursement frameworks. Such system‐level restrictions may differ from other regions, limiting the generalizability of these findings to healthcare systems with alternative drug access pathways or funding models.

Management strategies for oligoprogressive disease demonstrated comparable heterogeneity to that seen with second‐line systemic treatment strategies, highlighting the need for more standardized, consensus‐driven recommendations. Our data highlight the viability of multiple strategies, including radiotherapy and continued first‐line EGFR‐TKI therapy, in maintaining disease control. Among patients experiencing oligoprogression (*n* = 34), nearly half (*n* = 15) underwent radiotherapy at the progression site, in alignment with established clinical guidelines [20, 21, 22, 23, 42, 43]. Only a small subset (*n* = 4) required a concurrent switch in systemic therapy. Recently, the SABR‐COMET study [44], which included 18 patients with lung cancer (18% of the total cohort), demonstrated that stereotactic ablative radiotherapy significantly improved OS and PFS compared to standard of care alone, with no added toxicity supporting the role of local therapy in oligometastatic disease. The remaining patients in our study received alternative systemic therapies, including a switch to second‐line EGFR‐TKI or chemotherapy/immunotherapy. These findings highlight the need for standardized recommendations to guide clinical decision‐making and optimize patient outcomes in this evolving therapeutic landscape [20, 21, 22, 23, 45].

There are several emerging treatment strategies that are likely to reshape the treatment landscape in both first‐ and second‐line settings. The CHRYSALIS study [46] demonstrated clinical benefits of Amivantamab (EGFR‐MET bi‐specific antibody) and Lazertinib (third‐generation EGFR‐TKI) in patients with Osimertinib‐relapsed EGFR‐mutated NSCLC. Similarly, the MARIPOSA II study [47] showed that Amivantamab plus chemotherapy, with or without Lazertinib, improved PFS compared to chemotherapy alone in patients who had relapsed after prior Osimertinib therapy. The antibody‐drug conjugate (ADC) landscape is also rapidly evolving, with several agents showing early promise [48, 49], including the HER3‐targeted ADC patritumab deruxtecan, which has demonstrated durable responses and intracranial activity in pretreated Osimertinib‐relapsed patients in the HERTHENA‐Lung01 trial [50, 51]. In addition, HER2‐directed ADCs such as trastuzumab deruxtecan are expanding into NSCLC, while TROP2‐targeted agents like datopotamab deruxtecan and novel EGFR‐directed ADCs are also under active investigation [48, 49, 52, 53]. These emerging therapies offer strategies to overcome resistance, with potential considerations even for re‐biopsy of primary lesions or metastases to identify actionable targets. While these new therapies offer alternative treatment options, they also add complexity in sequencing decisions after first‐line EGFR‐TKI failure.

This study has several important limitations. The retrospective design introduces potential biases, including reliance on clinician‐determined progression rather than blinded review, which may have affected PFS estimates. Although the cohort represents the largest of its kind in Australia, subgroup numbers were small, limiting statistical power. Treatment delivery was heterogeneous and not protocolized, with decisions shaped by performance status, comorbidities, and access to therapies within Australia. All patients were recruited in New South Wales, where drug availability was determined by national reimbursement policies; notably, during the study period, second‐line Osimertinib was restricted to patients with confirmed T790M mutations, limiting generalizability to other health systems. Finally, EGFR mutation subtype data were unavailable, precluding assessment of outcomes by mutation type and limiting comparability with clinical trial cohorts, where treatment allocation was guided by mutational status. This is an important gap, particularly given evidence that exon 21 L858R mutations are associated with lower sensitivity and shorter duration of response and PFS compared with exon 19 deletions [54].

Despite its limitations, this study provides real‐world insights, capturing the complexity and variability of routine practice. The findings highlight substantial heterogeneity in second‐line treatment approaches and emphasize persistent gaps in care, reinforcing the urgent need for prospective clinical trials to guide therapeutic sequencing. With the treatment landscape continuing to evolve, the importance of such trials in informing future strategies will only increase.

## 5 Conclusion

Leveraging real‐world data from the EnRICH program, this study provides insights into the management of EGFR‐mutant mNSCLC, in the second‐line setting, in Australia, representing the largest retrospective analysis in this cohort. While the introduction of Osimertinib initially addressed the challenge of second‐line therapy, its use in earlier stages of disease and as first‐line treatment, together with the emergence of resistance, has reestablished the need for effective later‐line management options. In our cohort, first‐line treatment predominantly comprised early‐generation EGFR‐TKIs, and second‐line therapy similarly remained TKI‐focused; however, there was a noticeable shift toward Osimertinib following its approval and accessibility. Only a small subset of patients received chemotherapy or immunotherapy in the second‐line setting. Similar heterogeneity was observed in the management of oligoprogression, where continuation of first‐line EGFR‐TKI with radiotherapy to metastatic sites appeared to be a viable option in our cohort. These findings reflect the paucity of evidence in this area and highlight the uncertainty surrounding optimal second‐line treatment strategies, further emphasizing the need for standardized approaches and consensus guidelines.

Ongoing research is critical to refine treatment strategies and sequencing, particularly with the increasing availability of novel therapies. Further prospective studies and international collaborations are needed to optimize the management of EGFR‐driven mNSCLC, ultimately improving patient outcomes in this challenging area.

## 6 Clinical Practice Points

- EGFR‐driven mNSCLC accounts for around 10%–30% of all NSCLCs. EGFR‐TKIs are standard of care, in the first‐line setting, and have more recently become available in the second‐line setting. However, intrinsic or acquired resistance remains an ongoing concern, and there is an increasing need for effective therapeutic strategies. The scarcity of clinical trial data to guide subsequent lines of therapy highlights the importance of investigating real‐world data.
- In this study, leveraging real‐world data from The Embedding Research (and evidence) in Cancer Healthcare (EnRICH) program, we provide insights into the management of EGFR‐driven mNSCLC in Australia, representing the largest retrospective analysis in this cohort. Our cohort is uniquely placed as it includes patients pre and post, the availability of Osimertinib in the first‐ and second‐line settings.
- The study highlights significant heterogeneity in second‐line treatment strategies. However, Osimertinib remains the predominant choice following disease progression on early‐generation EGFR‐TKI therapy, while chemotherapy and immunotherapy are used less frequently. Additionally, switching between generations of EGFR‐TKI therapy emerged as a common strategy.
- The study also highlights the varied approaches to managing oligometastatic progression, emphasizing the need for consensus guidelines, with our findings supporting radiotherapy and continued first‐line EGFR‐TKI therapy as viable options for managing oligoprogression.
- Emerging therapies such as Amivantamab, Lazertinib, and ADCs have the potential to address the treatment gap beyond Osimertinib by overcoming resistance mechanisms and offering more durable options. At the same time, the expanding range of available treatments creates a complex therapeutic landscape, highlighting the need for more robust evidence to guide sequencing decisions and their integration into clinical practice.

## Author Contributions

**Bea Brown:** data curation, writing – review and editing. **Meghana Maddula:** conceptualization, methodology, data curation, formal analysis, writing – original draft, writing – review and editing.

## Funding

The authors have nothing to report.

## Ethics Statement

The study was conducted according to the guidelines of the Declaration of Helsinki, endorsed by the EnRICH Steering Committee, and approved by SLHD Lead HREC (RPA Zone) under protocol number X16‐0447 and 2019/ETH06379: Embedding Research (and evidence) in Cancer Healthcare (February 19, 2024).

## Consent

Informed consent was obtained from all subjects involved upon enrolment in the Embedding Research (and evidence) in Cancer Healthcare (EnRICH) Program.

## Conflicts of Interest

The authors declare no conflicts of interest.

## Supporting information


**Data S1:** cnr270428‐sup‐0001‐Supinfo.docx.

## Data Availability

The data that support the findings of this study are available from the corresponding author upon reasonable request.
