# Supplementary material for: Assessing Second‐Line Treatment Strategies and Outcomes in Epidermal Growth Factor Receptor (EGFR) Oncogene‐Driven Stage IV Non‐Small Cell Lung Cancer, Following a First‐Line EGFR‐TKI Therapy
Source: Cancer Rep (Hoboken). 2025 Dec 17;8(12):e70428. doi: 10.1002/cnr2.70428 (PMC12710434; doi:10.1002/cnr2.70428)
Supplement: Supplementary file 1 — Data S1: cnr270428‐sup‐0001‐Supinfo.docx. [file CNR2-8-e70428-s001.docx]

**TITLE PAGE**

**Assessing second-line treatment strategies and outcomes in epidermal growth factor receptor (EGFR) oncogene-driven stage IV non-small cell lung cancer, following a first-line EGFR-TKI therapy.**

**Running head:** **Treatment paths in EGFR-driven mNSCLC after TKI**

**Authors and Affiliations**

1. Meghana Maddula: University of New South Wales, Sydney, Australia; The Kinghorn Cancer Centre, Darlinghurst, Australia; St Vincent’s Hospital, Sydney, Australia. [m.maddula@unsw.edu.au](mailto:m.maddula@unsw.edu.au) [ORCID ID - 0009-0003-4819-9397]
2. Annelise Decaria: NHMRC Clinical Trials Centre, University of Sydney, NSW, Sydney, Australia; The University of Sydney, NSW, Sydney, Australia. [Annelise.decaria@sydney.edu.au](mailto:Annelise.decaria@sydney.edu.au) [ORCID ID – 0009-0005-2646-9311]
3. Bea Brown: NHMRC Clinical Trials Centre, University of Sydney, NSW, Sydney, Australia; The University of Sydney, NSW, Sydney, Australia. [bea.brown@sydney.edu.au](mailto:bea.brown@sydney.edu.au) [ORCID ID - 0000-0003-2851-1809]
4. John Simes: NHMRC Clinical Trials Centre, University of Sydney, NSW, Sydney, Australia; The University of Sydney, NSW, Sydney, Australia. [john.simes@sydney.edu.au](mailto:john.simes@sydney.edu.au) [ORCID ID - 0000-0002-3740-7563]
5. Michael Boyer: Chris O’Brien Lifehouse, Sydney, NSW, Australia. [michael.boyer@lh.org.au](mailto:michael.boyer@lh.org.au) [ORCID ID - 0000-0002-0452-2987]
6. Venessa Chin: The Garvan Institute of Medical Research, Sydney, Australia; The Kinghorn Cancer Centre, St Vincent's Hospital Sydney, Australia; University of New South Wales, Sydney, Australia. [v.chin@garvan.org.au](mailto:v.chin@garvan.org.au) [ORCID ID - 0000-0002-4630-4451]

**Short title:** EGFR-Driven Stage IV NSCLC: Second-Line Strategies and Outcomes

**Submitting/Corresponding Author Details**

- Dr Meghana Maddula
- Postal Address: 370 Victoria St, Darlinghurst NSW 2010
- Email: [m.maddula@unsw.edu.au](mailto:m.maddula@unsw.edu.au)
- Telephone: 0432710677

**Acknowledgements**

- This research did not receive any specific funding from public, commercial, or non-profit organisations
- This work was conducted in collaboration with the EnRICH Program, a flagship program of Sydney Catalyst, University of Sydney, NSW, Australia.
- We would like to thank Chris Brown from the NHMRC Clinical Trials Centre, University of Sydney for his valuable contributions to the statistical analyses in this study.
- We would also like to acknowledge Cure Cancer Australia, Lung Foundation Australia and St Vincent’s Clinical Research Foundation.

**SUPPLEMENTARY FIGURES AND TABLES**

***FIGURE S1. Flow diagram of patient inclusion***

***FIGURE S2. First-line treatment strategies***

***FIGURE S3. Second-line treatment strategies***

**Oscillate clinical trial_(39)_*

*ABCP = Atezolizumab + Bevacizumab + Carboplatin + Paclitaxel; CP = Carboplatin + Pemetrexed; CEA = Carboplatin + Etoposide + Atezolizumab; CPTD = Carboplatin + Pemetrexed + Tremelimumab + Durvalumab; CPP = Carboplatin + Pembrolizumab + Pemetrexed*

***FIGURE S4. Radiotherapy Strategies***

*ABCP = Atezolizumab + Bevacizumab + Carboplatin + Paclitaxel; CPP = Carboplatin + Pembrolizumab + Pemetrexed*

***TABLE S1. Australian Pharmaceutical Benefits Scheme Listing***

| ***FIRST-GENERATION EGFR-TKI*** | |
| --- | --- |
| ***Erlotinib*** | - 1 August 2008 – 2^nd^ line - monotherapy for patients with stage IIIB or IV NSCLC after prior platinum-based chemotherapy, where disease progression has occurred following docetaxel/pemetrexed or where these treatments are contraindicated or not tolerated - 1 Jan 2014 – 1^st^ line - monotherapy for stage IIIB or IV NSCLC with evidence of EGFR mutation |
| ***Gefitnib*** | - 1 July 2004 – 2^nd^ line - monotherapy, of locally advanced or mNSCLC, where disease progression has occurred following treatment with at least one chemotherapy agent and there is evidence that patient has EGFR mutation - 1 January 2014 – 1^st^ line - monotherapy for patients with Stage IIIB (locally advanced) or Stage IV (metastatic) NSCLC cancer with EGFR gene mutations |
| ***SECOND-GENERATION EGFR-TKI*** | |
| ***Afatinib*** | - 1 July 2018 – 1^st^ line/subsequent line – monotherapy for patients with stage IIIB or Stage IV NSCLC with EGFR gene mutation OR must have developed intolerance to another EGFR TKI |
| ***THIRD-GENERATION EGFR-TKI*** | |
| ***Osimertinib*** | - 1 Nov 2018 – 2^nd^ line – monotherapy for stage IV NSCLC with EGFR T790M gene mutation post-EGFR TKI - 1 Jan 2021 – 1^st^ line – monotherapy for stage IV NSCLC with EGFR gene mutation |

* eviQ Cancer Treatments Online 2020, Cancer Institute NSW, <https://www.eviq.org.au/>

** The Pharmaceutical Benefits Scheme (PBS), Australian Government (Department of Health and Aged Care), <https://www.pbs.gov.au>

***TABLE S2. Summary of key trial data versus outcomes from current study***

| ***STUDY*** | ***POPULATION*** | *TREATMENT* | ***PFS*** | ***OS*** | *NOTES* |
| --- | --- | --- | --- | --- | --- |
| ***Current study cohort*** | EGFR-mutant mNSCLC, post-EGFR-TKI | Overall cohort | **PFS 3.0 months** (95% CI, 1.32-5.49) | **OS 14.78 months** (95% CI, 13.24-23.66) | Real-world cohort (NSW, Australia) |
|  |  | TKI vs  Other treatment strategy | **PFS2 3.29 vs 1.74 months (HR 0.72, 95% CI 0.41-1.25)** | **OS 20.57 vs 9.99 months** (HR 0.68, 95% CI 0.40-1.17) |  |
| ***AURA3*** | T790m+ EGFR mut- mNSCLC, post-EGFR-TKI | Osimertinib  vs  Pemetrexed + Carboplatin/Cisplatin | **PFS 10.1 vs 4.4 months** (HR 0.30, 95% CI, 0.23-041) | **OS 26.8 vs 22.5 months** (HR 0.87, 95% CI, 0.67–1.12) | Phase III RCT |
| ***IMpower150*** | EGFR-mut mNSCLC, post-EGFR-TKI | Atezolizumab+Bevacizumab + Carboplatin+ Pemetrexed  vs Bevacizumab+Carboplatin+Pemetrexed |  | **OS 27.8 vs 18.1 months** (HR 0.74, 95% CI, 0.38-1.46) | Exploratory subgroup analysis of Phase III RCT |
